# Isolation, Antimicrobial Effect and Metabolite Analysis of *Bacillus amyloliquefaciens* ZJLMBA1908 against Citrus Canker Caused by *Xanthomonas citri* subsp. *citri*

**DOI:** 10.3390/microorganisms11122928

**Published:** 2023-12-06

**Authors:** Xinru Ke, Zilin Wu, Yucheng Liu, Yonglin Liang, Manling Du, Ya Li

**Affiliations:** College of Coastal Agricultural Science, Guangdong Ocean University, Zhanjiang 524088, China; kexinru@stu.gdou.edu.cn (X.K.); 2112204011@stu.gdou.edu.cn (Z.W.); 2112204073@stu.gdou.edu.cn (Y.L.); liangyonglin3@stu.gdou.edu.cn (Y.L.); dumanling@stu.gdou.edu.cn (M.D.)

**Keywords:** antagonistic bacteria, antimicrobial effect, metabolite analysis, biological control

## Abstract

Citrus canker caused by *Xanthomonas citri* subsp. *citri* is a devastating bacterial disease with severe implications for the citrus industry. Microorganisms possessing biocontrol capabilities against *X. citri* subsp. *citri* offer a highly promising strategy for healthy citrus management. In the present study, a broad-spectrum antagonist strain ZJLMBA1908 with potent antibacterial activity against *X. citri* subsp. *citri* was isolated from symptomatic lemon leaves, and identified as *Bacillus amyloliquefaciens*. Cell-free supernatant (CFS) of strain ZJLMBA1908 also exhibited remarkable antimicrobial activity, especially suppressing the growth of *X. citri* subsp. *citri* and *Nigrospora oryzae*, with inhibition rates of 27.71% and 63.75%, respectively. The antibacterial crude extract (CE) derived from the CFS displayed effective activity against *X. citri* subsp. *citri*. A preventive treatment using the CE significantly reduced the severity and incidence of citrus canker in a highly susceptible citrus host. Additionally, the CE maintained activity in the presence of protease and under a wide range of temperature and pH treatments. Applying high-performance liquid chromatography (HPLC) to separate and purify the CE resulted in the discovery of one highly potent anti-*X. citri* subsp. *citri* subfraction, namely CE3, which could completely inhibit the growth of *X. citri* subsp. *citri*. Liquid chromatography–electrospray ionization–mass spectrometry (LC–ESI–MS) analysis revealed that CE3 mainly consisted of palmitic acid, surfactin C15, phytosphingosine and dihydrosphingosine. Taken together, the results contribute to the possible biocontrol mechanisms of *B. amyloliquefaciens* ZJLMBA1908, as well as providing a promising new candidate strain as a biological control agent for controlling citrus canker.

## 1. Introduction

Citrus canker, resulting from infection by the Gram-negative bacterium *Xanthomonas citri* subsp. *citri*, is a disease of considerable distribution that affects most commercially cultivated citrus varieties [1]. Its impact mainly focuses on fruit, leaves and twigs, where lesions result in premature drop and reduced marketability, as well as impeding international fruit trade [2]. Currently, citrus canker is mainly managed using integrated management approaches such as planting pathogen-free certified trees, protecting orchards by installing windbreaks and spraying synthetic copper bactericides. The spray application of copper-based bactericides has been the primary counter-measure to prevent *X. citri* subsp. *citri* infections [3]. Nevertheless, owing to the prolonged utilization of copper agents, copper-resistant strains have emerged in *Xanthomonas* populations [4]. The residues of chemical bactericides also have caused substantial adverse effects on the environment. Therefore, the application of new environmentally friendly microbial source substances for the prevention and control of citrus canker disease has emerged as a current research hotspot.

Using beneficial microbe agents as biocontrols is an ecologically friendly alternative that provides effective measures to reduce the risk of plant pathogen resistance while being harmless to non-target species [5]. Over the past few decades, various antagonistic microorganisms have been identified as biological control agents (BCAs) for *X. citri* subsp. *citri*, such as the *Pseudomonas* spp. [6], *Bacillus* spp. [7], *Streptomyces* spp. [8], *Cronobacter* spp. and *Enterobacter* spp. [9].

Among the most promising BCAs, *Bacillus* species are harmless endophytic microorganisms that reside within plant tissues for a specific period without inducing symptoms of disease [10]. The application of cell suspensions or fermentations of *Bacillus* species as a means of controlling citrus diseases has garnered considerable attention worldwide. For instance, Daungfu et al. [11] reported that the leaves of lime plants inoculated with cell suspensions of *B. subtilis* LE24 or *B. amyloliquefaciens* LE109 significantly reduced the incidence of citrus canker. Chen et al. [12] proved that the culture filtrate of *B. amyloliquefaciens* DH-4 was capable of inhibiting the growth of *Penicillium digitatum* in vitro and suppressing the diameter of pathogen lesions in vivo. However, the effectiveness of cell suspensions or their fermentations in field applications still faces significant challenges due to their inherent lability, limited durability and susceptibility to environmental conditions [13,14]. 

In this study, a *Bacillus amyloliquefaciens* strain was isolated and identified from the endophytic bacteria of a symptomatic citrus and rice leaves from a local farm, and its inhibitory effects against some plant pathogens of southern China were evaluated in vitro. Subsequently, the potential biocontrol activity of the antimicrobial compounds produced by strain ZJLMBA1908 isolated from lemon leaves was evaluated against *X. citri* subsp. *citri*, and the main compounds responsible for the antibacterial function were identified. The findings of this research will facilitate the development of biological strategies to control citrus canker and provide an alternative to conventional copper treatment, which has caused serious environmental problems.

## 2. Material and Methods

### 2.1. Bacterial Strains and Plants

The five plant pathogenic fungi, namely *Fusarium oxysporum* f.sp. *cubense* race 4 (*Foc4*), *Neoscytalidium dimidiatum*, *Nigrospora oryzae*, *Fusarium solani* and *Sarocladium oryzae*, were kindly provided by Prof. Runhua Yi and Yuelian Liu of Guangdong Ocean University. *X. citri* subsp. *citri* strain zlm1908 (designated as *X. citri* subsp. *citri* zlm1908) was isolated from the mandarin cultivar Shatangju (*Citrus reticulata*) and preserved in our laboratory. The endophytic bacteria were isolated from lemon [*C. limon* (L.) Burm. F.] leaves from a lemon orchard that was infected with citrus canker and rice leaves from a rice field infected with bacterial leaf streak in the Zhanjiang district.

Three-year-old citrus trees (*Citrus sinensis* ‘Hongjiang’) were purchased from the Zhanjiang Huacheng Agriculture Development Company (Chikan District, Zhanjiang City, China), and grown in 20 cm × 30 cm pots in a greenhouse. The leaves of Hongjiang orange, which are susceptible to citrus canker, were used to perform the *X. citri* subsp. *citri* infection experiments.

### 2.2. Isolation and Purification of Endophytic Bacteria

Sterilization of the leaf surfaces and isolation of the endophytic bacteria were performed as described in Han et al. [15]. The leaf samples were disinfected with 70% ethanol, soaked in 2% sodium hypochlorite (NaClO) for 5 min, rinsed five times with sterilized water and finally drained. A 10 g sample of the sterilized leaves was weighed and cut into small fragments using sterile scissors, thoroughly pulverized using a sterile mortar and pestle and then added to a flask containing 90 mL of sterilized water, followed by shaking for 20 min to obtain suspension stock of the lemon leaves and rice leaves, respectively. Subsequently, a series of dilutions (10^−2^, 10^−3^, 10^−4^, 10^−5^ and 10^−6^) were prepared from the leaf debris suspension. Then, 100 μL of each diluted suspension was spread onto Luria-Bertani (LB) solid medium (tryptone 10 g/L, yeast extract 5 g/L, NaCl 10 g/L, pH 7.0) plates, respectively, and incubated at 32 °C. After 24 h, different-looking colonies were picked out onto fresh LB medium plates for pure culture preparation.

### 2.3. Screening for Antagonistic Bacteria against Plant Pathogens

Efficient broad-spectrum antagonist bacteria were screened using the plate confrontation method [16,17]. The strain *X. citri* subsp. *citri* and the five types of fungi were used as indicator microorganisms. For evaluating the antibacterial activity, the *X. citri* subsp. *citri* was cultured in the LB medium at 30 °C until reaching an optical density at 600 nm of 0.2. The culture was then evenly spread onto an LB agar plate and fresh endophytic bacteria were transferred onto the center of each plate and incubated at 30 °C for 48 h. For the antifungal activity assay, a 6 mm diameter agar plug of the indicator fungi was placed at the center of potato dextrose agar (PDA) (potato 200 g/L, glucose 20 g/L, agar 20 g/L, pH 7.0) plates (90 mm), and a fresh colony of endophytes was transferred onto the PDA dishes at approximately 25 mm from the fungal mycelia plug at four equidistant points. The control was PDA plates only inoculated with the pathogenic fungi. The plates were incubated at 28 °C until the control group reached 2/3 area of the plate. The antimicrobial activity was expressed in terms of the inhibition rate, which was calculated using the following formulas:

Inhibition rate (%) = R1/R2 × 100%,(1)

Inhibition rate (%) = (R3 − R1)/(R3 − 6) × 100%.(2)

‘R1’ represents the diameter of the inhibition zone, ‘R2’ represents the plate diameter, ‘R3’ represents the colony diameter of the plant pathogen in the control and ‘6’ represents the diameter of the fungal mycelia plug. Formula (1) is used for the indicator bacteria *X. citri* subsp. *citri*, while Formula (2) is used for the indicator fungi.

### 2.4. Identification of Strain ZJLMBA1908 

To identify the isolated strain, strain ZJLMBA1908 with the strongest broad-spectrum antagonistic activity was inoculated onto the LB plates at 32 °C. Gram staining was detected during an incubation period of 18–24 h, while an observation of the culture characteristics was conducted at 2 d. Physiological and biochemical characterizations, including catalase activity, starch hydrolysis, a Voges–Proskauer reaction, a methyl red test, gelatin liquefaction, phenylalanine deaminase activity, anaerobic culture, growth at 3 °C, carbon source utilization and salt tolerance, were conducted following *Bergey’s Manual of Determinative Bacteriology* and the common bacterial identification manual [18,19].

Afterward, strain ZJLMBA1908 was further identified via phylogenetic analysis of the sequences of the 16S rDNA gene, gyrase gene (*gyrA*) and RNA polymerase β subunit gene (*rpoB*). The genomic DNA of strain ZJLMBA1908 was isolated and purified using the Bacteria DNA Kit [Accurate Biotechnology (Hunan) Co., Ltd., Changsha, China]. The 16S rRNA was amplified via PCR using the primers 27 F (5′-AGAGTTTGATCMTGGCTCAG-3′) and 1492 R (5′-TACGGYTACCTTGTTACGACTT-3′) [20]. The *gyrA* gene was amplified using the primers *gyrA*-F (5′-CAGTCAGGAAATGCGTACGTCCTT-3′) and *gyrA*-R (5′-CAAGGTAATGCTCCAGGCATTGCT-3′) [21]. The *rpoB* gene was amplified using the primers *rpoB*-F (5′-AGGTCAACTAGTTCAGTATGGAC-3′) and *rpoB*-R (5′-AAGAACCGTAACCGGCAACTT-3′) [22]. The PCR amplification system (50 μL) contained 3 μL of DNA template, 25 μL of 2 × Ex Taq Master Mix [Accurate Biotechnology (Hunan) Co., Ltd., Changsha, China], 1 μL each of the forward and reverse primers and 20 μL of Rnase-free water. The PCR conditions were as follows: 94 °C for 2 min; 35 cycles of 94 °C for 30 s, 58 °C for 30 s and 72 °C for 1 min; and a final extension at 72 °C for 10 min. The amplified products were resolved using 1% agarose gel electrophoresis; afterward, the gel was stained with ethidium bromide, photographed and analyzed using a gel documentation system; the PCR products were subsequently purified and sequenced by Sangon Biotech (Shanghai) Co., Ltd. (China). The obtained sequences were compared via homology alignment analysis using the BLAST program on the NCBI website and the FASTA sequences of the three genes were submitted to GenBank. The *gyrA* and *rpoB* gene sequences were aligned using Clustal X (2.0) and then spliced using EditPlus Text Editor v3.70 (290). Phylogenetic trees were constructed using the neighbor-joining method using the MEGA 11.0 software and the clade stability of the resulting tree was assessed using bootstrap analysis with 1000 replicates.

### 2.5. Preparation and Antimicrobial Activity Determination of ZJLMBA1908 Cell-Free Supernatant (CFS)

The cell-free supernatant (CFS) containing the secondary metabolites of strain ZJLMBA1908 was collected following the method of Li et al. [23] with slight modifications. Strain ZJLMBA1908 was preincubated in LB medium at 37 °C, 180 rpm until OD_600nm_ reached 0.1. Then, the 1% inoculum (*v*/*v*) was transferred into a new 50 mL LB medium and incubated under the same conditions. After incubating for 24 h, the supernatant of culture was harvested and centrifuged at 13,535× *g* for 10 min at 4 °C, and then filtered through a 0.22 μm microporous filter to obtain the CFS.

The antimicrobial activity of the CFS was assayed using Oxford cup tests for strain *X. citri* subsp. *citri* and dual culture for the five pathogenic fungi [24]. The sterile LB medium containing 0.1% of the indicator bacteria *X. citri* subsp. *citri* (OD_600nm_ = 0.2) was poured onto sterile plates (90 mm). Of the prepared CFS, 75, 175, and 275 μL was, respectively, added to the Oxford cups placed on the medium containing the indicator bacteria. Subsequently, the plates were incubated at 30 °C for 48 h. Then, 15 mL of the PDA medium including 150, 300, 600, 1200, and 2400 μL of the prepared CFS was poured onto sterile plates, then an indicator fungi of the 6 mm diameter plug was placed in the center of the plates and incubated at 28 °C until the control group covered 2/3 of the area of the plate. The treatment without CFS addition was used as a control and the inhibition rate to evaluate the antimicrobial abilities was calculated as described in Section 2.1.

### 2.6. Preparation and MIC/MBC Determination against X. citri *subsp.* citri of Crude Extract (CE) from ZJLMBA1908 CFS

The crude extract (CE) was obtained from the ZJLMBA1908 CFS according to the method of Im et al. [25]. Ethyl acetate was added in an equal volume to the CFS, and the upper organic phase was collected after stratification. Thereafter, it was dried under a vacuum using Sy-2000 spinning vapor at 60 °C, dissolved in 1 mL of PBS buffer or methanol and filtered through a 0.22 μm microporous filter. Solutions dissolved in PBS were utilized for the minimum inhibitory concentration (MIC), minimum bactericidal concentration (MBC), and greenhouse experiments and stability assessments, while methanol solutions were employed for the HPLC and LC–MS assays.

To determine the MIC of the CE against *X. citri* subsp. *citri* analysis, various concentrations of the CE were subject to two-fold serial dilution in sterile broth ranging from 0.09 to 369.50 μg/mL in 48-well plates [26]. Each well contained 1000 μL CE and 1000 μL *X. citri* subsp. *citri* culture (1 × 10^7^ CFU/mL). The negative control was composed of undiluted CE and LB medium, while a positive control included LB medium inoculated with *X. citri* subsp. *citri*. The 48-well plate covered with a plastic lid was incubated at 30 °C and 180 rpm for 24 h. The absorbance of the reaction solution was measured at 600 nm using a spectrophotometer. The MIC was defined as the lowest concentration at which the observable growth of the indicator bacteria was completely suppressed. Thereafter, the MBC was assayed by spreading 100 μL of the cultures from each well onto an LB plate and incubating it at 30 °C for 72 h. The MBC was defined as the lowest concentration that killed any indicator bacterial growth in the LB plates following a 72 h incubation period at 30 °C.

### 2.7. Biocontrol Assays of CE under Greenhouse Conditions

Susceptible citrus hosts of Hongjiang orange were used to assess the biocontrol potential of the CE under greenhouse conditions using the spraying inoculation method [27]. Symptomatic leaves of a consistent color, maturity and size were randomly selected and washed with tap water, followed by sterilization with 75% ethanol, and rinsed with sterile water. In accordance with the prophylactic protocol, the leaves were subsequently categorized into three groups: leaves receiving *X. citri* subsp. *citri* cell suspension spraying (positive control), leaves sprayed with both the PBS buffer and CE (negative control) and leaves sprayed with both the CE and *X. citri* subsp. *citri* bacterial suspension. Overnight cultures of *X. citri* subsp. *citri* (1 × 10^7^ CFU/mL) were centrifuged at 13,535× *g* for 10 min, collected, resuspended in PBS buffer and adjusted to 1 × 10^7^ CFU/mL. The leaves were then sprayed with 300 μL of the CE at concentrations according to the MBC (184.75, 369.5 and 739 μg/mL) on their surface, followed by 300 μL of *X. citri* subsp. *citri* cell suspension. After drying, the leaves were covered with plastic bags for 3 days at 25 °C and maintained at 90% relative humidity. Five citrus leaves were selected in each treatment. Each experiment was carried out for three plants. At 30 days post-inoculation, the inhibition rate was assessed based on the method described by de Oliveira et al. [28], with certain modifications. The inhibition rate was recorded by counting the number of spots on the sprayed leaves using the following formula:

Inhibition rate (%) = (N1 − N2)/N2 × 100%,(3)

‘N1’ represents the number of spots on the diseased leaves of the positive control and ‘N2’ represents the number of spots on the treated leaves.

### 2.8. Determination of CE Stability to Thermal, pH and Proteinase Enzymes

The CE was dissolved with PBS to obtain a final concentration of 184.75 μg/mL. Firstly, the CE was heated at 37 °C, 60 °C, 80 °C and 100 °C in a water bath for 30 min, and then heated at 121 °C in an autoclave at a pressure of 0.105 MPa for 30 min. The thermal stability of the CE was then evaluated after cooling to room temperature [17]. Thereafter, the pH of the CE was adjusted to 2–12 with 1 mol/L HCl and 1 mol/L NaOH to explore its pH stability [29]. After incubating the above samples at 37 °C for 3 h, their pH was adjusted to the original pH (7.28) to measure the antibacterial activity. For the enzyme sensitivity test, the CE was separately mixed with different proteases, including proteinase K (pH 7.5), trypsin (pH 7.5) and pepsin A (pH 2.0), to a final protease concentration of 1 mg/mL. The mixture was incubated at 37 °C for 2 h, followed by termination via boiling for 5 min [30]. During these experiments, the untreated CE was used as the control, and the antibacterial activities against the *X. citri* subsp. *citri* were evaluated using the Oxford cup method, as described in Section 2.5.

### 2.9. Purification of CE Using HPLC

The CE was purified using preparative high-performance liquid chromatography (HPLC) and an Agilent 1260 Infinity II Preparative LC System (Agilent Technologies, Inc., Santa Clara, CA, USA) equipped with a Zorbax Eclipse XDB-C18 (9.4 × 250 mm, 5 μm, Agilent Technologies, Inc., Santa Clara, CA, USA) column. The mobile phase was composed of 0.1% formic acid in water (A) and acetonitrile (B) with a flow rate of 1 mL/min, and the effluent was monitored at a UV wavelength of 230 nm. Gradient elution was performed with the following program: 0–1 min, 0–2% B; 1–9 min, 2–50% B; 9–20 min, 50–98% B; 20–25 min, 98% B; 25–35 min, 98–2% B; 35–40 min, 0% B. The eluate was collected in three time periods (9–15 min, 15–25 min, 25–34 min) and each fraction was assayed for antagonistic activity against *X. citri* subsp. *citri* using colony-counting methods. Specifically, the fractions were evaporated to remove acetonitrile via rotary evaporation, redissolved in methanol, mixed with LB medium at 50 °C and poured into blank plates. The control was the same volume of methanol mixed with LB. The *X. citri* subsp. *citri* (OD_600nm_ = 0.2) diluted to 10^−5^ was applied to the plates above, incubated at 32 °C for 48 h and the number of colonies was counted to evaluate the growth inhibition. Subsequently, the eluted fractions with the best antibacterial activity were identified using liquid chromatography–electrospray ionization–mass spectrometry (LC–ESI–MS).

### 2.10. Identification of CE Using LC–ESI–MS 

LC–ESI–MS analyses of the fractions with the best antibacterial activity were performed on a hybrid quadrupole time-of-flight tandem mass spectrometer equipped with Turbo V sources and a Turbo ion spray interface (AB SCIEX TripleTOF^®^ 5600+, AB SCIEX Pte. Ltd., Concord, ON, Canada). Liquid chromatography was applied on a Sepax GP-C18 column (1.8 µm, 120 Å, 2.1 mm × 150 mm) and the column temperature was maintained at 40 °C. The mobile phase was composed of 0.1% formic acid in water (A) and acetonitrile (B) with a flow rate of 0.3 mL/min. The gradient elution conditions were as follows: 0 min, 5% B; 0–10 min, 5–70%; 10 to 17 min, 70–100% B; 17–18 min, 100% B; 18–19 min, 100–5% B; 19 to 21 min, 5% B. Mass spectrometric data were recorded in positive and negative ion modes with injection voltages of 5500 V and 4400 V, ion source temperatures of 500 °C and 450 °C and a mass scan range of 100–1200 Da. The collision gas used was nitrogen, and the fragment energy was set at 35 ± 15 eV. The obtained LC–MS raw data were processed using the MS-DIAL 5.1 software to select possible adducts, peak alignment, deconvolution and putative metabolite identification based on MSE experiments [31]. The match score was automatically generated using MS-DIAL and the main antibacterial substances with a match score >1.0 were selected via comparison with existing data in the database.

### 2.11. Statistical Analysis

All experiments were repeated in triplicate under the same conditions. Statistical analysis of the data was carried out using SPSS 22.0 (SPSS Inc., Chicago, IL, USA). Duncan’s multiple-range test and one-way analysis of variance (ANOVA) were employed to assess the significance of the differences, with *p* < 0.05 indicating significant differences.

## 3. Results

### 3.1. Antagonistic Effect of Different Endophytic Bacteria

**Figure 1:**
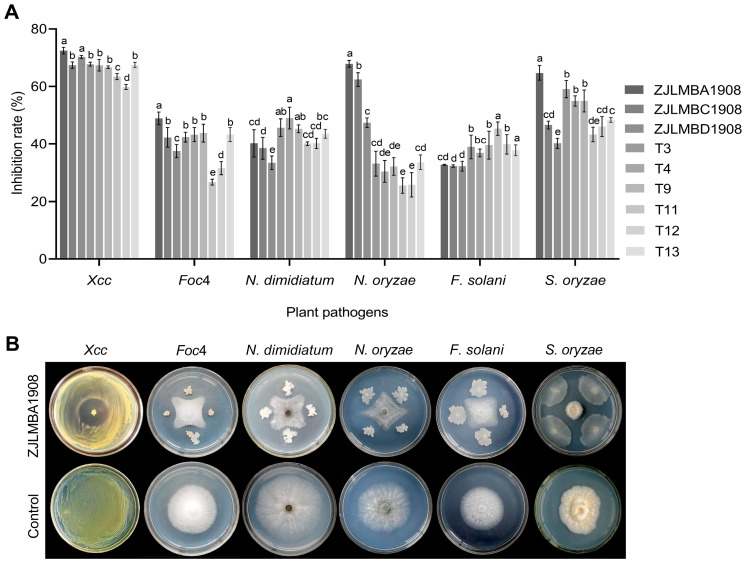
In vitro antagonistic effect of nine endophytic bacteria against six phytopathogens. (**A**) The inhibition rate of nine endophytic bacteria against six phytopathogens. Error bars indicate standard errors of the means from three repeated experiments. Different letters indicate a significant difference according to the Duncan’s multiple-range test (*p* < 0.05); (**B**) Antagonistic effect of strain ZJLMBA1908 against six phytopathogens.

A total of 52 bacterial strains were isolated from the lemon and rice leaves using the gradient dilution method, and 9 strains (ZJLMBA1908, ZJLMBC1908, ZJLMBD1908, T3, T4, T9, T11, T12 and T13) showed good antagonistic activity using the plate confrontation method; the results are shown in Figure 1A. The antagonistic activity of the nine strains against the target pathogen displayed significant variation. Via comparison with the other eight antagonistic bacteria, strain ZJLMBA1908 demonstrated a strong antimicrobial effect and its inhibition rates against *X. citri* subsp. *citri*, *Foc4*, *N. oryzae* and *S. oryzae* were, respectively, 36.33%, 48.90%, 67.91% and 64.52% (Figure 1B). For the plant pathogens *N. dimidiatum* and *F. solani*, although the inhibition rate was not the highest among the nine antagonists, strain ZJLMBA1908 also showed antibacterial potential with inhibition rates of 40.20% and 32.75%. Therefore, strain ZJLMBA1908 exhibited a broad spectrum against plant pathogens and was chosen for further investigation.

### 3.2. Identification of Strain ZJLMBA1908

**Figure 2:**
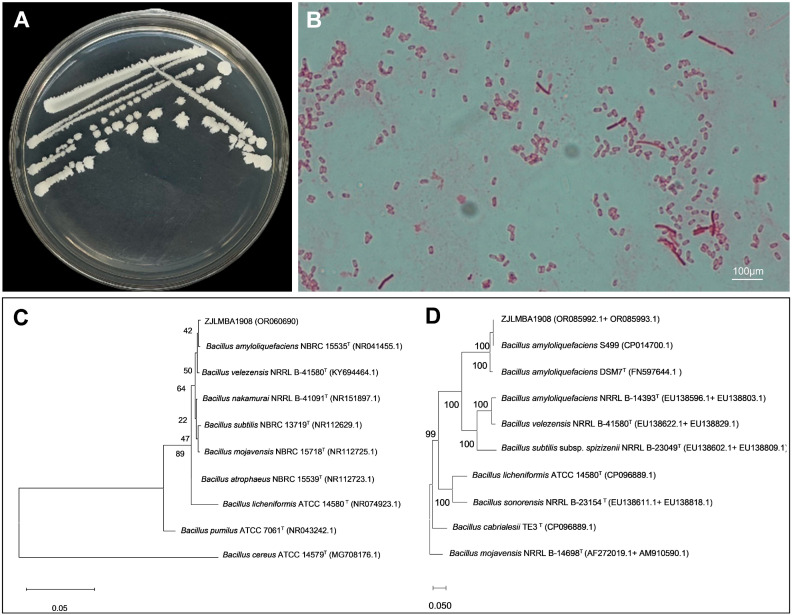
Identification of strain ZJLMBA1908. (**A**) Colony morphology of strain ZJLMBA1908 on LB solid medium; (**B**) cellular morphology of strain ZJLMBA1908 with Gram-positive staining; (**C**) phylogenetic tree of ZJLMBA1908 based on 16S rDNA gene sequence; (**D**) phylogenetic tree of ZJLMBA1908 based on joining the sequences of *gyrA* and *rpoB* genes. The scale bar represents the number of substitutions per base position and the letter T represents a type strain.

The colonies of strain ZJLMBA1908 were white, round or oval with irregular-shaped edges and a rough, opaque surface on the LB medium (Figure 2A). Microscopically, the bacteria appeared rod-shaped with motility, with a size of (0.7–0.8) μm × (1.5–2.5) μm, produced oval endospores and exhibited the characteristics of a Gram-positive bacterium (Figure 2B). Thereby, they were shown to be a *Bacillus* sp. The physiological and biochemical characteristics of strain ZJLMBA1908 are documented in Table 1. Positive reactions were observed for the catalase activity test, starch hydrolysis, Voges–Proskauer test, methyl red test and gelatin liquefaction, while negative reactions were found in phenylalanine deaminase, anaerobic culture and 3 °C growth, and there was no salt tolerance at 20% (Table 1). Additionally, sugar and alcohol fermentation experiments demonstrated that strain ZJLMBA1908 could ferment mannose, xylose, glucose, sucrose, galactose and sorbitol. The strain was found to have characteristics similar to those of *B. amyloliquefaciens*.

The 16S rRNA gene, *gyrA* gene and *rpoB* gene sequences were submitted to the GenBank database with accession numbers OR060690, OR085992 and OR085993. Phylogenetic tree analysis of the 16S rRNA gene sequence showed that strain ZJLMBA1908 shared the closest genetic relationship with *B. amyloliquefaciens* NBRC15535 (Figure 2C). This result was further confirmed using a phylogenetic tree constructed from the sequences of two genes (*gyrA* and *rpoB*), which showed that *B. amyloliquefaciens* S499 and strain ZJLMBA1908 clustered to a single branch (Figure 2D). In conclusion, the strain ZJLMBA1908 isolated from lemon leaves was identified as *B. amyloliquefaciens*.

### 3.3. Antagonistic Effect Analysis of ZJLMBA1908 CFS

**Figure 3:**
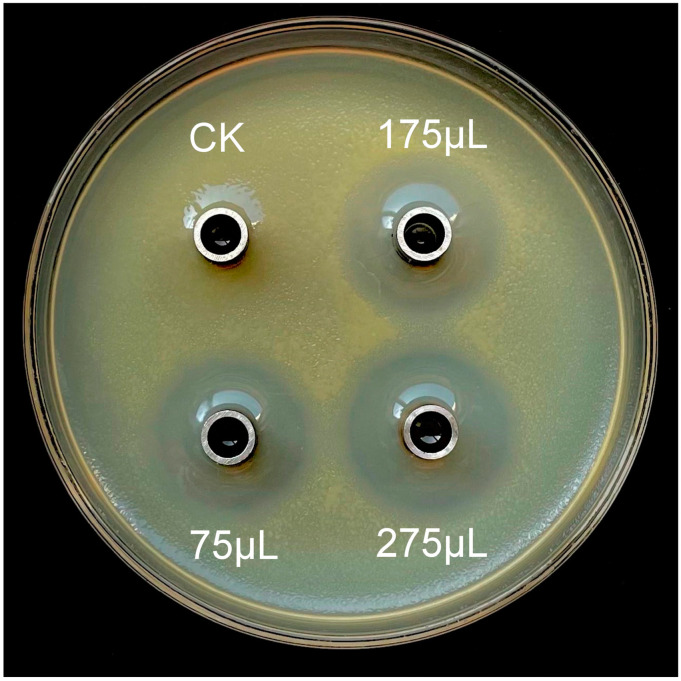
Antibacterial effect of various volumes of CFS (cell-free supernatant) obtained from *B. amyloliquefaciens* ZJLMBA1908 against *X. citri* subsp. *citri*. The CK (control check) group was treated with sterile water.

**Figure 4:**
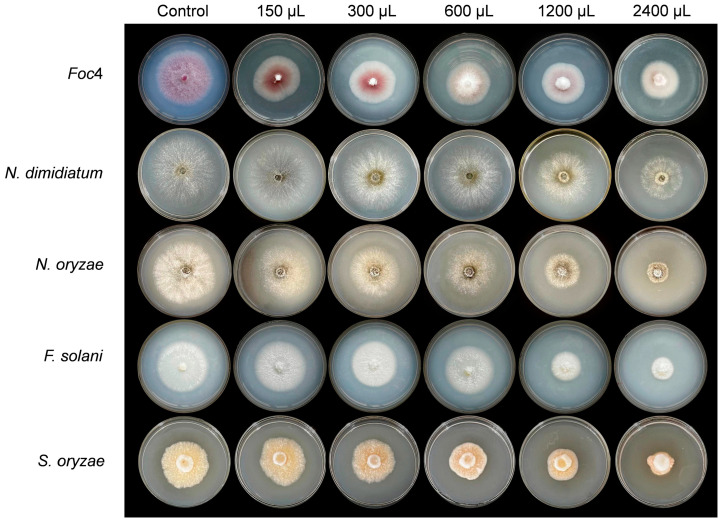
Antagonistic effect of various volumes of CFS (cell-free supernatant) obtained from *B. amyloliquefaciens* ZJLMBA1908 against five pathogenic fungi.

Various amounts of the CFS produced by strain ZJLMBA1908 were added to the medium to assess its antagonistic action against plant pathogens. The outcomes indicated that the CFS substantially suppressed the growth of all tested pathogens by increasing the dosage of the treatment (Figure 3 and Figure 4) and showed superior antagonistic effects compared to the strain itself. For *X. citri* subsp. *citri*, the inhibition rate increased with an increase in the CFS volume from 25.19% to 27.71% (Figure 3 and Table 2). For five different plant pathogen fungi, when the volume of the CFS increased from 150 µL to 2400 µL, the CFS showed antifungal activity with inhibition rates ranging from 17.41% to 33.72%, 2.90% to 40.32%, 4.25% to 63.75%, 1.97% to 53.86% and 6.35% to 39.79% against *Foc4*, *N. dimidiatum*, *N. oryzae*, *F. solani* and *S. oryzae*, respectively (Figure 4 and Table 3). Therefore, the CFS of strain ZJLMBA1908 contained potent antimicrobial compounds.

### 3.4. The MIC and MBC of Crude Extract (CE) against X. citri *subsp.* citri

**Figure 5:**
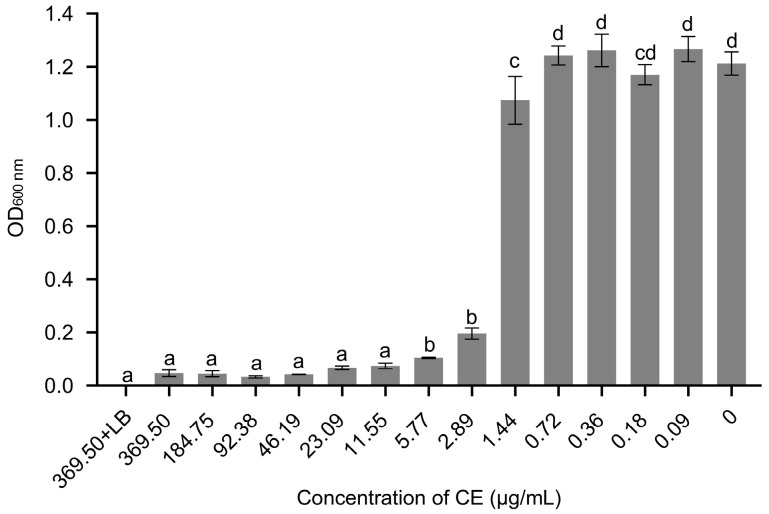
The OD_600 nm_ of *X. citri* subsp. *citri* after being treated with various concentrations of CE (crude extract) obtained from *B. amyloliquefaciens* ZJLMBA1908 in culture. Error bars indicate standard errors of the means from three repeated experiments. Different letters indicated a significant difference according to Duncan’s multiple-range test (*p* < 0.05).

An effective concentration of CE against *X. citri* subsp. *citri* was evaluated by determining its MIC and MBC values. The results indicate that when cultured in an LB medium containing 11.55 μg/mL of the CE, there was no significant difference in optical density compared to the negative control (Figure 5). Additionally, the absence of visible pathogen growth was observed with increasing concentrations, suggesting nearly complete inhibition of *X. citri* subsp. *citri* growth. Cell suspensions treated with the CE at concentrations ranging from 11.55 to 369.50 μg/mL were then transferred to agar plates and the growth was observed after 72 h. No colony formation was detected on the plates for suspensions treated with the CE at concentrations ≥184.75 μg/mL. Hence, the MIC and MBC values of the CE against *X. citri* subsp. *citri* were 11.55 and 184.75 μg/mL, respectively.

### 3.5. Evaluation of Biocontrol Efficiency of CE

**Figure 6:**
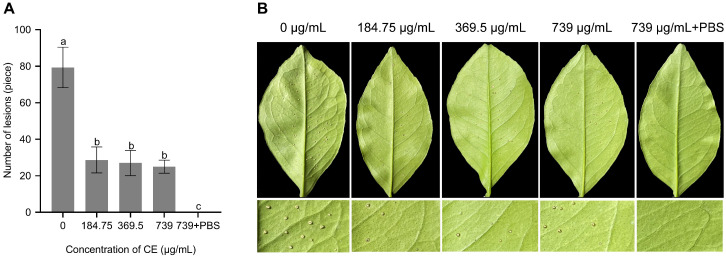
Biological control efficiency of CE (crude extract) obtained from *B. amyloliquefaciens* ZJLMBA1908 on *X. citri* subsp. *citri* under greenhouse conditions. (**A**) Quantification of canker lesions in citrus leaves; (**B**) the canker disease symptoms were treated with different concentrations of CE after 30 days post inoculation. Error bars indicate standard errors of the means from three repeated experiments. Different letters indicate a significant difference according to Duncan’s multiple-range test (*p* < 0.05).

After 30 days of inoculation, the foliage inoculated with pathogens exhibited severe raised and suberized lesions, whereas that treated with the CE had a noteworthy decrease in canker lesions. The symptoms of typical canker disease gradually decreased with increasing CE spray concentration, with 62.28%, 64.47% and 67.11% inhibition when applied at 184.75, 369.5 and 740 μg/mL, respectively (Figure 6A). Furthermore, leaves treated with the CE displayed minimal visual damage with a slight increase in diameter and only slightly raised pustules at the inoculation site. In contrast, positive control leaves tended to show massive lesions with large, round spots lifted to form a corky appearance (Figure 6B). The results showed effective leaf protection against *X. citri* subsp. *citri* infection and the prevention of further citrus canker development using foliar spraying of the CE.

### 3.6. CE Stability Analysis

**Figure 7:**
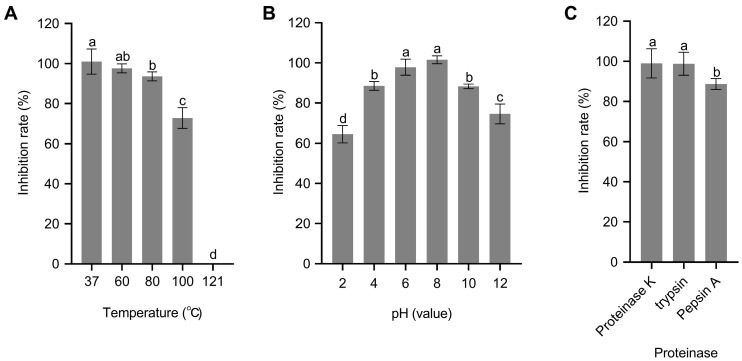
Stability of CE (crude extract) obtained from *B. amyloliquefaciens* ZJLMBA1908 under various conditions. (**A**) CE was treated at a wide range of temperatures; (**B**) CE was treated at a wide range of pHs; (**C**) CE was treated using different proteases. Standard deviations of three independent experiments are represented by error bars. Error bars indicate standard errors of the means from three repeated experiments. Different letters indicate a significant difference according to Duncan’s multiple-range test (*p* < 0.05).

The CE had remarkable thermal stability, with over 70% inhibitory activity after exposure to temperatures of 37 °C, 60 °C, 80 °C and 100 °C for 30 min, while a significant loss of activity was observed at 121 °C (Figure 7A). Regarding the pH stability, the CE was stable over a wide range of pH values. In particular, the antimicrobial activity of the CE remained above 85% of the original activity between pHs of 4 and 10, with a gradual decrease observed when the pH was below 2 or above 10, but still maintaining inhibition rates of 64.52% and 74.54%, respectively (Figure 7B). Additionally, the CE also displayed excellent resistance to protease degradation, being insensitive to proteinase K and trypsin and only slightly sensitive to pepsin A (Figure 7C). These results implied that the CE possesses a wide range of activity, enabling it to be adaptable to various environmental conditions to effectively inhibit pathogenic microorganisms, and it might be utilized as a biocontrol agent in agriculture.

### 3.7. Analysis of the Antibacterial Activity of the Purified CE

**Figure 8:**
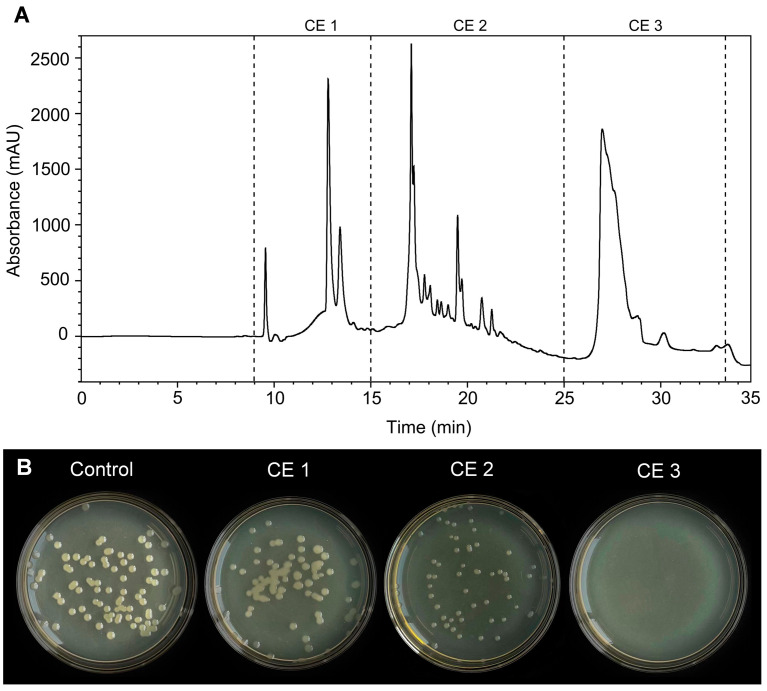
The antibacterial assay of purified CE (crude extract) obtained from *B. amyloliquefaciens* ZJLMBA1908 eluted fractions against *X. citri* subsp. *citri*. (**A**) The collected CE eluted fractions with different elution times in total ion current chromatogram; (**B**) the antagonistic activity of each fraction against *X. citri* subsp. *citri*.

The CE was separated and purified using HPLC, and obtained fractions with elution times of 9–34 min (sample numbers CE1–3) were further determined for antibacterial activity. Among these fractions, CE3 exhibited the strongest antagonistic activity against *X. citri* subsp. *citri* with 100% inhibition, as evidenced by the absence of growing colonies (Figure 8B). In contrast, CE1 and CE2 displayed low inhibition rates, amounting to 32.48% and 35.88%, respectively. Therefore, the CE3 fraction was collected and subjected to further mass spectrometric analysis to identify the active compounds.

### 3.8. Identification of Antibacterial Components from CE3

The LC–ESI–MS analysis revealed that CE3 of strain ZJLMBA1908 contained a variety of different organic compounds, including fatty acids, lipopeptides, sphingosine, phenols, flavonoids, alkaloids, cinnamic acid, steroids, terpenoids and esters (Table 4). Palmitic acid (36.46%), surfactin C15 (5.25%), dihydrosphingosine (4.28%) and phytosphingosine (2.58%) were the predominant components of CE3 in strain ZJLMBA1908. These findings suggest that fatty acids, lipopeptides and sphingosine may be the main metabolites of ZJLMBA1908 responsible for its anti-*X. citri* subsp. *citri* effects.

## 4. Discussion

Citrus canker caused by *X. citri* subsp. *citri* infects virtually all major citrus varieties, posing a severe threat to citrus production [32]. Biological control, employing microorganisms and their products, has proven effective in managing plant diseases caused by various pathogens. In contrast to conventional pesticides, biological agents are regarded as sustainable alternatives that pose no harm to mammals and exhibit minimal environmental impact [33]. In this research, the *B. amyloliquefaciens* strain ZJLMBA1908 was isolated and identified from lemon leaves. The strain and the cell-free supernatant (CFS) of this strain both exhibited antagonistic activity against *X. citri* subsp. *citri* and five pathogenic fungi, including *Foc4*, *N. dimidiatum*, *N. oryzae*, *F. solani* and *S. oryzae*. The crude extract (CE) obtained from the CFS of strain ZJLMBA1908 displayed strong biocontrol efficacy against *X. citri* subsp. *citri* in in vitro and in vivo experiments. The main anti-*X. citri* subsp. *citri* compounds in ZJLMBA1908 were identified as fatty acids, lipopeptides and sphingosine. These findings suggest that *B. amyloliquefaciens* ZJLMBA1908 and its cell extract could be employed as a biocontrol agent against *X. citri* subsp. *citri* and several fungal pathogens.

*B. amyloliquefaciens* is an antagonistic strain with demonstrated potential antimicrobial activities [34]. The *B. amyloliquefaciens* strain ZJLMBA1908 and its corresponding CFS exhibited remarkable and variable levels of antagonistic effects against the targeted plant pathogens in this study, probably because this strain can fine-tune its antagonistic strategy by producing different antibiotics depending on the nature of the different pathogens. Supporting evidence can be found in previous studies where *B. amyloliquefaciens* SQR9 modified antifungal substance production against six different soil-borne fungal pathogens [35]. *B. amyloliquefaciens* PGPBacCA1 was identified to have surfactin and iturin in the liquid culture, whereas for the solid medium, an additional class of lipopeptide called fengycin was identified within the bacterial–fungal growth inhibition zone of strain PGPBacCA1 [36].

Moreover, the biocontrol effects of *B. amyloliquefaciens* against citrus canker caused by *X. citri* subsp. *citri* were studied. The CE effectively inhibited the growth of *X. citri* subsp. *citri* in vitro, exhibiting low MIC values (11.55 μg/mL) compared to related studies. Rabbee et al. [37] reported MIC values of 46.9 μg/mL for the ethyl acetate CFS extract of endophytic *B. velezensis* against *X. citri* subsp. *citri*, while Nugroho et al. [38] reported that the MIC of *Staphylococcus pasteuri* B1 and *Staphylococcus warneri* C8 CFSs against *X. citri* subsp. *citri* was 25 μg/mL. The application of the CE also resulted in a substantial reduction in the occurrence of citrus canker in the host plants, achieving an approximate 60% reduction compared with those in the control. A single applied dose of 184.75 μg/mL of the CE was able to control *X. citri* subsp. *citri* infection in the citrus leaves, and no significant differences were observed between different applied doses. However, a trend emerged between the dosage of the CE and its effectiveness, with higher dosages corresponding to superior efficacy, suggesting that it could be meaningful to employ elevated dosages of the CE to control highly developed infections of *X. citri* subsp. *citri*. Conversely, for emerging infections, a lower dose of the BCA should suffice to exert adequate control measures. Additionally, the CE maintained stable activities in broad temperature and pH ranges, as well as in the presence of protease. These findings indicate that the anti-*X. citri* subsp. *citri* metabolites in the filtrate exhibited stable antagonistic properties, with similarity to the investigations of Nugroho et al. [38]. It also implies that the practical utilization of the CE in natural settings would not be affected by fluctuations in environmental factors.

The available evidence substantiates a correlation between the biocontrol efficacy of *B. amyloliquefaciens* and its capacity to synthesize a diverse array of antimicrobial compounds [39]. Wang et al. [40] reported *B. amyloliquefaciens* CE’s association with *X. citri* subsp. *citri* inhibition was likely due to the action of surfactin, fengycin and iturin, detected using electrospray ionization–mass spectrometry analysis of the culture extracts. However, the LC–ESI–MS analysis identified fatty acids, lipopeptides, sphingosine, alkaloids, cinnamic acid, flavonoids, phenols, esters and steroids as the main anti-*X. citri* subsp. *citri* substances produced by *B. amyloliquefaciens*. Among the anti-*X. citri* subsp. *citri* compounds, fatty acids, particularly palmitic acid, were identified as the most abundant main compounds produced by ZJLMBA1908. This suggests that long-chain fatty acids have a major influence on the anti-*X. citri* subsp. *citri* activity. Lin et al. [41] demonstrated linoleic acid, as a long-chain fatty acid, was found to be a potent compound against *X. citri* subsp. *citri* and it highly suppressed the growth of *X. citri* subsp. *citri* by disrupting the integrity of the cell membrane and inducing excessive production of reactive oxygen species (ROS). Lipopeptides, including surfactin A, surfactin B, surfactin C and maribasin B, dominated over the other groups. While the potent antibacterial activity of surfactin is widely acknowledged, maribasin B was identified for the first time as possessing antibacterial properties in *B. amyloliquefacien* [42]. Prior work by Zhang et al. [43] reported the broad-spectrum antifungal activity of maribasin B secreted by *B. marinus* against phytopathogens. Meanwhile, dihydrosphingosine and phytosphingosine were the other main components in strain ZJLMBA1908’s crude extract. These compounds mainly act as long-chain sphingoid bases, with reports of their potent anti-microbial activity inhibiting the growth of several yeast and fungal species in vitro [44]. Researchers have indicated the biological control potential of phenols, flavonoids, alkaloids, cinnamic acid, steroids, terpenoids and esters [45,46,47,48,49,50]. Thus, these compounds could altogether contribute to the antibacterial activity of *B. amyloliquefaciens* ZJLMBA1908. Although the exact mechanism of the antibacterial activity of these substances against *X. citri* subsp. *citri* has not been determined, the prime target of their action may be bacterial cell membranes, potentially disrupting processes essential for cellular defense and functionality.

## 5. Conclusions

In this study, a broad-spectrum antagonist, *B. amyloliquefaciens* ZJLMBA1908, was isolated and demonstrated a potent antibacterial ability against *X. citri* subsp. *citri*. The cell-free supernatant (CFS) obtained from strain ZJLMBA1908 also exhibited significant antimicrobial activity against a range of plant pathogens, including *X. citri* subsp. *citri*, *Foc4*, *N. dimidiatum*, *N. oryzae*, *F. solani* and *S. oryzae.* Application of the crude extract (CE) derived from the CFS remarkably restrained the growth of *X. citri* subsp. *citri* and reduced the severity of citrus canker symptoms. Using HPLC and LC–ESI–MS to analyze CE3 (separated from the CE), the main anti-*X. citri* subsp. *citri* active components of strain ZJLMBA1908 were identified as palmitic acid, phytosphingosine, dihydrosphingosine and surfactin C15. Furthermore, the CE maintained stable activities in a wide range of temperatures and pHs and in the presence of protease. These results indicate that the strain ZJLMBA1908 had great potential as a promising biocontrol agent for controlling the citrus canker caused by *X. citri* subsp. *citri*.

## Figures and Tables

**Table 1:** Physiological and biochemical characteristics of strain ZJLMBA1908.

Characteristics	Results	Characteristics	Results
Catalase activity	+	D-mannose	+
Starch hydrolysis	+	D-xylose	+
Voges–Proskauer	+	D-Glucose	+
Methyl red	+	Sucrose	+
Gelatin liquefaction	+	D-galactose	+
Phenylalanine deaminase	−	Salt tolerance test with 0.1% NaCl	+
Anaerobic culture	−	Salt tolerance test with 5% NaCl	+
3 °C growth	−	Salt tolerance test with 10% NaCl	+
Sorbitol	+	Salt tolerance test with 20% NaCl	−

Note: “+”, is Positive; “−”is Negative.

**Table 2:** Inhibition diameter and inhibition rate of various volumes of CFS (cell-free supernatant) obtained from *B. amyloliquefaciens* ZJLMBA1908 against *X. citri* subsp. *citri*.

CFS (µL)	*X. citri* subsp. *citri*	
	Inhibition Diameter (mm) ^1,2^	Inhibition Rate (%) ^1,2^
75	22.67 ± 0.38 ^b^	25.19 ± 0.01 ^b^
175	24.38 ± 0.69 ^ab^	27.08 ± 0.01 ^ab^
275	24.94 ± 0.31 ^a^	27.71 ± 0.00 ^a^

^1^ Numerical values are mean ± SD of triplicates. ^2^ Means were tested using Duncan’s multiple-range test. Means followed by the same letter are not significantly different (*p* < 0.05) within the same column.

**Table 3:** Inhibition rate of various volumes of CFS (cell-free supernatant) obtained from *B. amyloliquefaciens* ZJLMBA1908 against five pathogenic fungi.

CFS (µL)	Inhibition Rate (%) ^1,2^				
	*Foc4*	*N. dimidiatum*	*N. oryzae*	*F. solani*	*S. oryzae*
150	17.41 ± 0.48 ^d^	2.90 ± 1.09 ^d^	4.25 ± 0.15 ^e^	1.97 ± 0.55 ^e^	6.35 ± 0.19 ^e^
300	21.94 ± 0.36 ^c^	8.52 ± 0.42 ^c^	7.89 ± 0.09 ^d^	6.33 ± 0.51 ^d^	9.35 ± 0.03 ^d^
600	25.10 ± 0.64 ^b^	10.93 ± 1.76 ^c^	17.62 ± 0.58 ^c^	11.12 ± 0.43 ^c^	17.15 ± 1.05 ^c^
1200	25.82 ± 0.72 ^b^	24.29 ± 0.32 ^b^	40.66 ± 0.20 ^b^	42.13 ± 0.65 ^b^	30.22 ± 0.45 ^b^
2400	33.72 ± 0.67 ^a^	40.32 ± 0.13 ^a^	63.75 ± 0.76 ^a^	53.86 ± 1.62 ^a^	39.79 ± 1.08 ^a^

^1^ Numerical values are mean ± SD of triplicates. ^2^ Means were tested using Duncan’s multiple-range test. Means followed by the same letter are not significantly different (*p* < 0.05) within the same column.

**Table 4:** Chemical composition of CE (crude extract) obtained from *B. amyloliquefaciens* ZJLMBA1908 eluted fraction CE3.

Group	Metabolite	Ion Type	Measured (*m*/*z*)	Molecular Formula	Peak Area
Fatty acids	Palmitic acid	[M−H]^−^	255.2428	C_16_H_32_O_2_	7,943,267.400
	Pentadecanoic acid	[M−H]^−^	241.2269	C_15_H_30_O_2_	527,287.553
	Linoleic acid	[M−H]^−^	279.2437	C_18_H_32_O_2_	324,303.341
	Petroselinic acid	[M−H]^−^	281.2605	C_18_H_34_O_2_	284,596.411
	Myristic acid	[M−H]^−^	277.2086	C_14_H_28_O_2_	259,333.257
	Docosahexanoic acid	[M−H]^−^	327.2402	C_22_H_32_O_2_	72,556.094
	α-Linolenic acid	[M−H]^−^	277.2271	C_18_H_30_O_2_	32,681.762
Lipopeptides	Surfactin C	[M+H]^+^	1036.687	C_53_H_93_N_7_O_13_	505,074.195
	Surfactin B	[M+Na]^+^	1044.651	C_52_H_91_N_7_O_13_	70,432.186
	Surfactin C	[M+Na]^+^	1058.671	C_53_H_93_N_7_O_13_	69,917.200
	Surfactin A	[M+H]^+^	1008.656	C_51_H_89_N_7_O_13_	63,120.98
	Maribasin B	[M+H]^+^	1057.564	C_49_H_76_N_12_O_14_	37,023.161
Sphingosine	Dihydrosphingosine	[M+H]^+^	302.3047	C_18_H_39_NO_2_	411,920.009
	Phytosphingosine	[M+H]^+^	318.2997	C_18_H_39_NO_3_	247,879.301
Phenols	2,6-di-tert-butyl-4-methylphenol	[M−H]^−^	219.1839	C_15_H_24_O	563,053.549
	5-caffeoylquinic acid	[M+Na]^+^	377.0894	C_16_H_18_O_9_	20,313.456
Flavonoids	Procyanidin A1	[M+H]^+^	577.1314	C_30_H_24_O_12_	219,703.147
	Glabrol	[M+H]^+^	393.2101	C_25_H_28_O_4_	92,746.922
	Genistein	[M+H]^+^	271.0595	C_15_H_10_O_5_	25,979.231
Alkaloids	Agelasine	[M+NH_4_]^+^	475.3233	C_26_H_40_ClN_5_	214,877.055
	Vedelianin	[M+H]^+^	481.2607	C_29_H_36_O_6_	72,421.615
	Okaramine J	[M+H]^+^	525.2859	C_32_H_36_N_4_O_3_	49,245.988
Steroids	Lithocholic acid	[M−H]^−^	375.2863	C_24_H_40_O_3_	304,811.408
Cinnamic acid	Cinnamaldehyde	[M+H]^+^	133.0651	C_9_H_8_O	35,660.410
	4-methoxycinnamic acid	[M−H]^−^	177.0638	C_10_H_10_O_3_	117,098.160
Terpenoids	Cucurbitacin B	[M+Na]^+^	581.3050	C_32_H_46_O_8_	28,239.383
	Ginsenoside Rk2	[M+H]^+^	605.4328	C_36_H_60_O_7_	26,324.767
Esters	Lovastatin	[M+Na]^+^	427.2459	C_24_H_36_O_5_	19,164.271

## Data Availability

The data that support the findings of this study are available from the corresponding author upon reasonable request.
